# Effect of Graphene on the Performance of Silicon–Carbon Composite Anode Materials for Lithium-Ion Batteries

**DOI:** 10.3390/ma17030754

**Published:** 2024-02-04

**Authors:** Chengyuan Ni, Chengdong Xia, Wenping Liu, Wei Xu, Zhiqiang Shan, Xiaoxu Lei, Haiqing Qin, Zhendong Tao

**Affiliations:** 1 Key Laboratory of Air-Driven Equipment Technology of Zhejiang Province, Quzhou University, Quzhou 324000, China; nichengyuan1@126.com (C.N.); x1669686944@163.com (W.X.); 221122020426@zjut.edu.cn (Z.T.); 2 Guangxi Key Laboratory of Superhard Material, National Engineering Research Center for Special Mineral Material, Guangxi Technology Innovation Center for Special Mineral Material, China Nonferrous Metal (Guilin) Geology and Mining Co., Ltd., Guilin 541004, China; leixiaoxu2006@126.com (X.L.); qinhaiqing5218@163.com (H.Q.); 3 School of Materials Science and Engineering, Guangxi Key Laboratory of Information Materials, Guilin University of Electronic Technology, Guilin 541004, China; 4 School of Environmental and Food Engineering, Liuzhou Vocational & Technical College, Liuzhou 545000, China; shanzq1977@163.com

**Keywords:** nano-Si, graphite, graphene, Li-ion batteries, electrochemical performance

## Abstract

(Si/graphite)@C and (Si/graphite/graphene)@C were synthesized by coating asphalt-cracked carbon on the surface of a Si-based precursor by spray drying, followed by heat treatment at 1000 °C under vacuum for 2h. The impact of graphene on the performance of silicon–carbon composite-based anode materials for lithium-ion batteries (LIBs) was investigated. Transmission electron microscopy (TEM) and selected area electron diffraction (SAED) images of (Si/graphite/graphene)@C showed that the nano-Si and graphene particles were dispersed on the surface of graphite, and thermogravimetric analysis (TGA) curves indicated that the content of silicon in the (Si/graphite/graphene)@C was 18.91%. More bituminous cracking carbon formed on the surface of the (Si/graphite/graphene)@C due to the large specific surface area of graphene. (Si/Graphite/Graphene)@C delivered first discharge and charge capacities of 860.4 and 782.1 mAh/g, respectively, initial coulombic efficiency (ICE) of 90.9%, and capacity retention of 74.5% after 200 cycles. The addition of graphene effectively improved the cycling performance of the Si-based anode materials, which can be attributed to the reduction of electrochemical polarization due to the good structural stability and high conductivity of graphene.

## 1. Introduction

Recently, LIBs have attracted considerable attention as a promising power source for various applications, such as portable electronics and electric vehicles, due to their greater energy density, higher operating voltages, lower self-discharge, and maintenance requirements compared to other rechargeable batteries [1,2]. Graphite is a commercial anode material for LIBs, as it has a long cycle life, high coulombic efficiency, and quite a low cost [3,4]. However, its low specific lithiation capacity (372 mAh/g) is unable to meet the increasing demands of secondary batteries. Silicon (Si) anode material is one of the most promising alternatives for graphite, since its high theoretical capacity of 3579 mAh/g for Li_15_Si_4_ is about 10 times higher than that of graphite [5]. However, the disadvantages of the Si anode are low electrical conductivity and large volume changes on cycling, which leads to fast capacity fading and a poor cycle life [6,7,8]. Many studies have reported techniques for preparing silicon/carbon (Si/C) composites with different graphite or carbon precursors, which is an effective approach to suppress the severe capacity degradation of pure Si [9,10,11,12]. While Si is dispersed into the carbonaceous matrix, the Si/C composite can buffer the large volume expansion of Si and improve its conductivity [13,14,15,16]. Consequently, Si/C composites exhibit better reversible capacity and cycling performance. For example, silicon@carbon (Si@C) composites with an embedded structure were prepared by Chen et al. Silicon nanoparticles and carbon shells formed a stable solid electrolyte interface (SEI) film during the lithiation and delithiation processes at the working electrode. Si@C composites were successfully prepared by spray drying and subsequent calcination with a reversible capacity of 1033.70 mAh/g after 100 cycles at a current density of 1 A/g [17].

To address the low conductivity of Si, various studies report introducing conductive coating layers on the Si surface, which can effectively increase the conductivity of Si and improve overall electrochemical performance [18,19]. For instance, a composite material with a porous mesophase pitch double-layer carbon structure of coated silicon nanoparticles (Si@C@PMP) was fabricated by coating the Si nanoparticles with mesophase pitch-ordered soft carbon, which exhibit a reversible capacity of 774 mA h/g for 300 cycles at 0.2 A/g [20]. A Si-based composite with a double carbon layer (Si@C@C) coating was achieved using high-temperature pyrolysis and mechanical stirring, which significantly improved the electrochemical performance of the Si anode [21]. In our earlier research, a large-scale approach was designed to prepare a Si-based composite with a graphite@nano-Si coated with carbon pitch, and after 300 cycles, the graphite@nano-Si@C composite’s capacity retention was 66.03%. [21]. These approaches enhance the conductivity of the anode material by coating the silicon surface with an amorphous carbon layer.

Graphene is similar to the carbon matrix for nano-Si particles, which is important in the energy conversion field due to its superior electronic conductivity, excellent structural flexibility, and large specific surface area. When used as anode materials for lithium-ion batteries, graphene’s electrochemical energy storage performance is better than that of graphite, and its charging speed is faster than that of graphite, which is expected to achieve fast charging. In addition, high-current-discharge-capacity lithium-ion batteries will also be improved [2,22]. For anode materials, graphene has both the ability to provide good electron transport channels and excellent lithium-ion transport properties. However, graphene is difficult to compound directly with spherical nanosilicon, and silicon and graphene are usually dispersed on a graphite matrix [23,24]. Su et al. [25] prepared a Si/graphite@graphene by spray drying followed by heating treatment, and the sample exhibited a high initial charge capacity of 827 mAh/g at 100 mA/g. However, the capacity retention rate of the Si/graphite@graphene was only about 60% at 100 mA/g after 50 cycles, which still needs to be further improved for industrial requirements. 

Herein, (Si/graphite)@C and (Si/graphite/graphene)@C were prepared by coating asphalt-cracked carbon on the nano-Si surface and a carbon precursor by spray drying followed by heat treatment at 1000 °C under vacuum for 2 h. The impact of graphene on the electrochemical performance and cycle stability of silicon–carbon composite-based anode materials for LIBs were investigated.

## 2. Experiment

### 2.1. Sample Preparation

Nano-Si was prepared in Tekna’s 15 kW induction plasma system using micron-sized Si powder (Shanghai Aladdin Biochemical Technology Co., Ltd., Fengxian District, Shanghai, China, specification or purity: 99.9%, 1–3 μm) as the raw material, with a previously reported preparation method [21]. Graphite powders (Fangda Carbon New Materials Co., Ltd., Lanzhou, China) exhibited a reversible capacity of 345 mAh/g. The preparation of (Si/graphite/graphene)@C was carried out as follows: nano-Si powder, graphene (Shanghai Aladdin Biochemical Technology Co., Ltd, Fengxian District, Shanghai, China; specification or purity: ink, 40%; solids: 100 g; viscosity: 5.5 Pa.s.), graphite, and methylcellulose (Shanghai Aladdin Biochemical Technology Co., Ltd., Fengxian District, Shanghai, China; specification or purity: 1500 mPa.s.), with a weight ratio of 20:5:73:2, were dispersed in deionized water by ultrasonication with mechanical stirring. Then, the uniformly dispersed liquid was spray-dried (Shanghai shunyi Tech Co., Ltd., Jiading District, Shanghai, China) to obtain a homogeneous precursor power. Next, the weight ratio of the precursor and asphalt powders (supplier: Hebei Hankai Energy Science and Technology Development Co., Ltd., Handan, China; CAS No. 8052-42-4; fineness: 0–3 mm, 80–100 mesh; fixed carbon: ≥40%) was set to 9:1, and the obtained mixture was added into a hot reactor and stirred for hot coating at 600 °C for 2 h and cooled to room temperature. Finally, the asphalt-coated precursor powers were taken into a vacuum furnace for heat treatment at 1000 °C under vacuum for 2 h to obtain the (Si/graphite/graphene)@C. The preparation of (Si/graphite)@C was similar to that mentioned above, with a nano-Si powder, graphite, and methylcellulose at a weight ratio of 20:78:2.

### 2.2. Sample Characterization

The crystalline phases of synthesized composites were characterized by X-ray diffraction (XRD; D8 ADVANCE; BrukerAXSGmbH, Baden-Württemberg, Germany) scanning in the range of 20° to 100°with scan rate 8°/min. Transmission electron microscopy (TEM; JEM-2100F; Nidec Corporation, Tokyo, Japan) and field emission electron microscopy (FE-SEM; Quanta 200; Thermo Fisher Scientific, Waltham, MA, USA) were used to study the composites’ microstructure and morphology. The content of Si in the synthesized material was verified by thermogravimetric analysis and differential scanning calorimetry (DSC).

### 2.3. Electrochemical Measurements

The 80 wt% (Si/graphite/graphene)@C and (Si/graphite)@C were used as the active materials, 10 wt% Super P (TIMCAL) as the conductive agent, and 10 wt% mixture solution of butadiene styrene rubber and carboxymethyl cellulose (Chengdu indigo power sources Co., Ltd., Chengdu, China, weight ratio 3:2) as the binder. The above three substances were added to the container, and deionized water was added and stirred to obtain 35 wt%. slurry. The slurry was coated onto Cu foil and dried for 8 h at 80 °C with vacuum tube test furnace to obtain test electrode.

The 2032 coin half-cell was assembled using the as-obtained 12 mm wafer electrode as the working electrode, with lithium metal foil as the counter electrode, Celgard 2400 as the separator, and 1 M LiPF_6_ in EC:EMC:DMC (1:1:1, *v*/*v*) as the electrolyte. At 100 mA/g to 0.01 V and then 10 mA/g to 0.005 V, discharge capacity was assessed. Charge capacity was assessed at 100 mA/g to 1.5 V, and cycle performance was repeatedly monitored. Electrochemical impedance spectroscopy (EIS) was carried out in the frequency range of 100 KHz to 0.01 Hz, using a Solartron 1287 electrochemical Workstation.

## 3. Results and Discussion

### 3.1. Materials Characterization 

**Figure 1:**
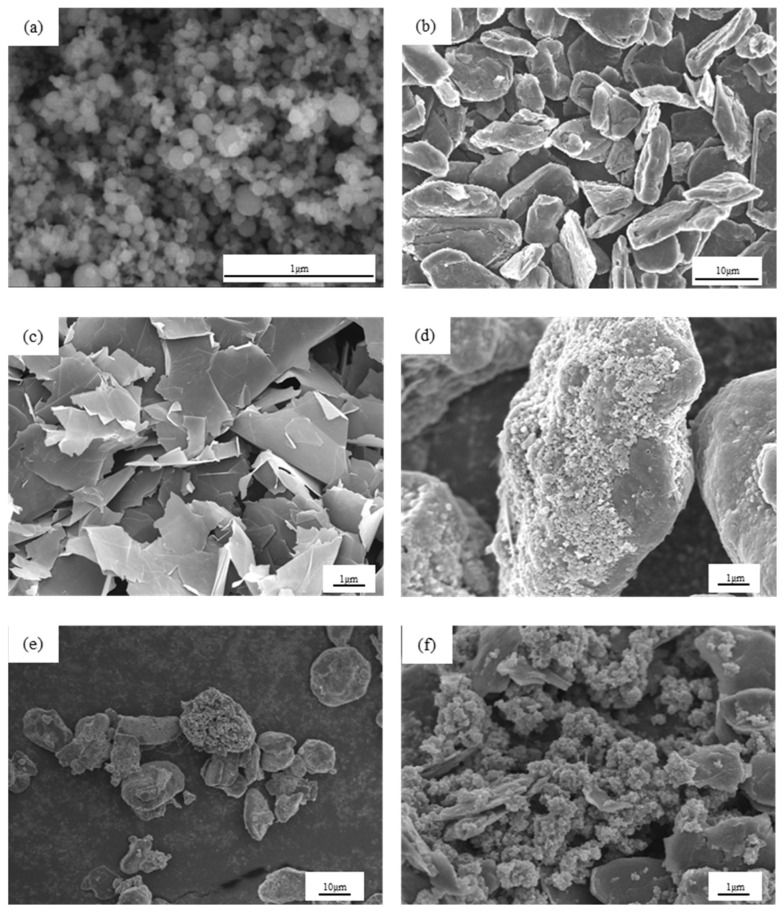
SEM images of (**a**) nano-Si, (**b**) graphite, (**c**) graphene, (**d**) (Si/graphite)@C composite, and (**e**,**f**) (Si/graphite/graphene)@C composite.

SEM images of (Si/graphite)@C and (Si/graphite/graphene)@C are shown in Figure 1. Figure 1a shows the morphology of silicon nanoparticles, which have a spherical structure with a nanometer diameter. Graphite has a lamellar structure with multiple particles stacked together with a particle size distribution between 5 and 20 µm and an average diameter of 8.46 µm, as shown in Figure 1b. Figure 1c shows the morphology of graphene, which has a lamellar structure. Figure 1d shows the morphology of (Si/graphite)@C with nano-Si particles distributed directly on the graphite particle surface. Nano-Si particles have a spherical morphology without obvious agglomeration and are randomly linked together due to surface tension and electrostatic forces. Figure 1e,f shows the morphology of (Si/graphite/graphene)@C, wherein nano-Si particles are mixed with graphene and are distributed on the surface of graphite particles, with some particles glued together under the action of bituminous cracking carbon, which contributes to the electrical conductivity of the material. Graphite particles are irregularly shaped with accidented surface, which is beneficial to loading nano-Si particles.

**Figure 2:**
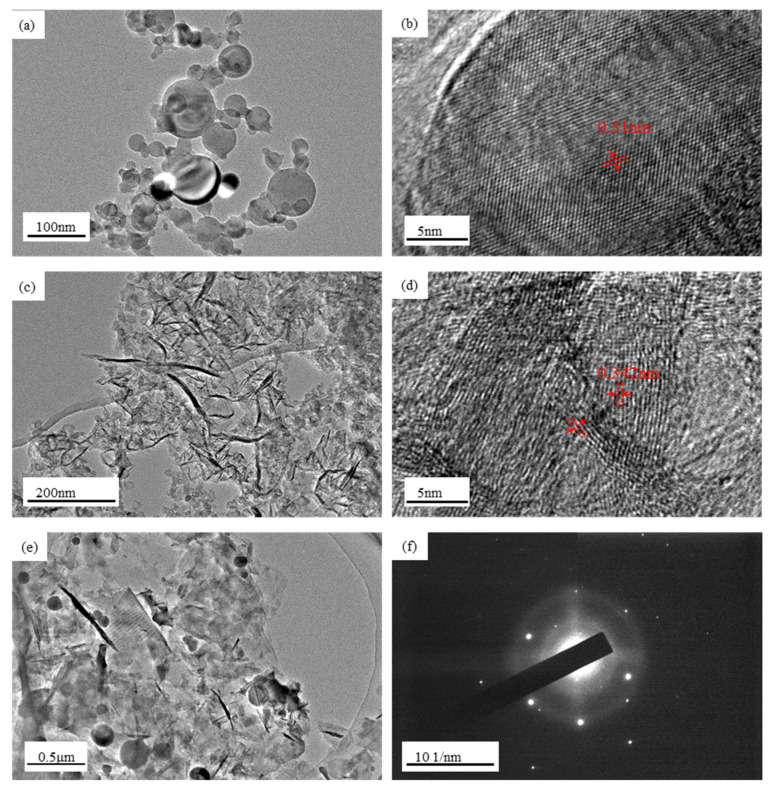
TEM images of (**a**,**b**) nano-Si, (**c**,**d**) graphene, and (**e**) (Si/graphite/graphene)@C composite; (**f**) SAED image of (Si/graphite/graphene)@C composite.

The TEM image of Si nanoparticles in Figure 2a,b shows spherical nanoparticles with varying sizes and single crystals of planar arrangement. In a single particle, the spacing between adjacent planes is about 0.31 nm, which corresponds to the (111) crystallographic plane of Si. Nanosized silicon particles can effectively alleviate the particle pulverization caused by the volume expansion of silicon and shorten the diffusion distance of lithium ions, thus improving the cycling stability performance of the electrode. However, volume expansion during charging and discharging leads to uneven distribution of stress at the interface of the composite material, resulting in poor cycling performance at high multiplicity. Graphene is distributed in a chain pattern with a polycrystalline arrangement, as shown in Figure 2c,d. TEM and SAED images of (Si/graphite/graphene)@C are shown in Figure 2e,f, respectively. The electron diffraction spot and electron diffraction ring in Figure 2f correspond to nanocrystalline Si and polycrystalline graphite structures, respectively. Graphene and nano-Si particles were well mixed, and the anode material’s electrical conductivity was increased due to the presence of graphene flakes, preventing the sample from making contact with the electrolyte. Moreover, the sufficient mixing of graphene and silicon nanoparticles alleviates the uneven stress distribution caused by the volume expansion of silicon and improves the cycling performance of the anode material at high multiplicity.

**Figure 3:**
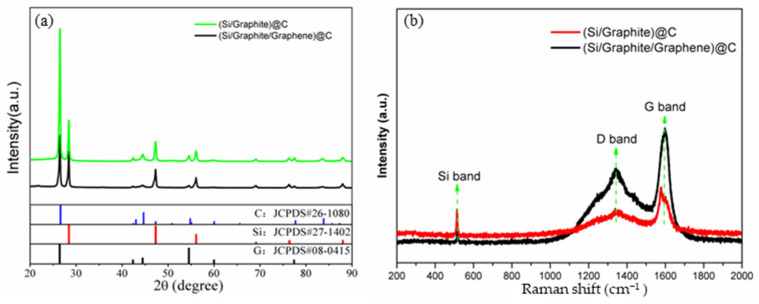
(**a**) XRD pattern and (**b**) Raman spectra of (Si/graphite)@C and (Si/graphite/graphene)@C composite.

The structural characteristics of (Si/graphite)@C and (Si/graphite/graphene)@C were analyzed by XRD, as shown in Figure 3a. Carbon (JCPDS# 26-1080), crystalline graphite (JCPDS# 08-0415), and cubic Si (JCPDS# 27-1402) phases can be found in the main diffraction peaks [21]. The cubic Si phase corresponds to nano-Si; the carbon phase is mainly bituminous cracking carbon, while the crystalline graphitic phase is attributed to graphite and graphene. Moreover, there are no peaks of SiO_2_ or SiC in the XRD patterns, indicating that nano-Si is not oxidized or does not react with carbon during material preparation [26,27]. The Raman spectra in Figure 3b were further used to verify the structure of the composites. The sharp peak at 514 cm^−1^ is assigned to crystalline Si, while the peaks at 1347 cm^−1^ and 1596 cm^−1^ correspond to amorphous carbon (D band) and ordered structure graphitic carbon (G band), respectively [21,28,29]. The intensity of the D and G bands in (Si/graphite/graphene)@C are higher than those of (Si/graphite)@C, mainly due to the addition of graphene. The ordered structure of graphene results in the increase in the intensity of G band in (Si/graphite/graphene)@C. There is more bituminous cracking carbon formed on the surface of (Si/graphite/graphene)@C due to the large specific surface area of graphene; consequently, the intensity of the D band increases.

**Figure 4:**
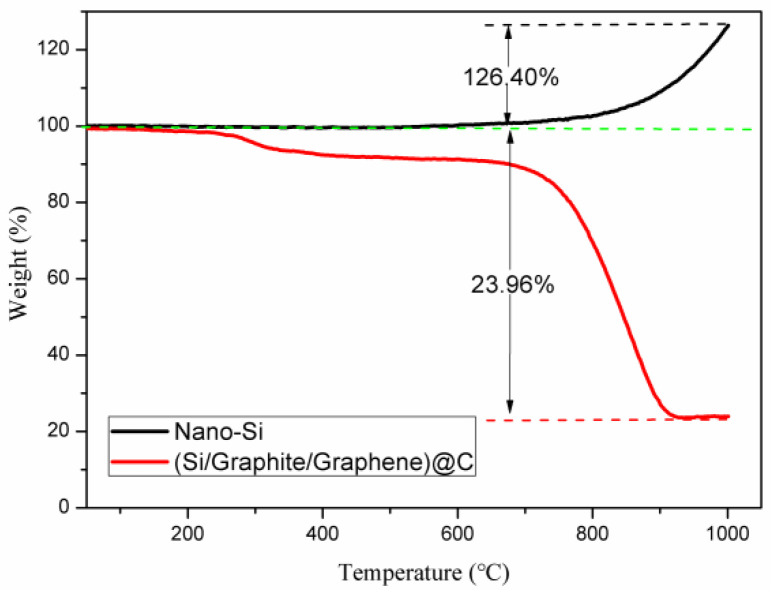
TGA curves of nano-Si and (Si/graphite/graphene)@C composite.

The Si content in (Si/graphite/graphene)@C was confirmed via the TGA curves of nano-Si and (Si/graphite/graphene)@C carried out in air. When heated to 1000 °C, Si was oxidized to SiO_2_, while graphite, graphene, and pitch carbon were oxidized to CO_2_ gas. Thus, the specific values of (Si/graphite/graphene)@C and nano-Si from the TGA curve (as shown in Figure 4) indicate silicon content in (Si/graphite/graphene)@C. The weight of nano-Si increased by 126.40%, while the weight of (Si/graphite/graphene)@C decreased by 23.96%. Thus, the Si content in (Si/graphite/graphene)@C could be calculated as 18.91% (23.96%/126.40%). The theoretical specific capacity of silicon is 3579 mAh/g. Multiplying the weight share of silicon by the theoretical specific capacity of silicon gives a maximum gram-specific capacity of 676.79 mAh/g (3579 × 18.91%) provided by silicon in (silicon/graphite/graphene)@C.

### 3.2. Electrochemical Properties

**Figure 5:**
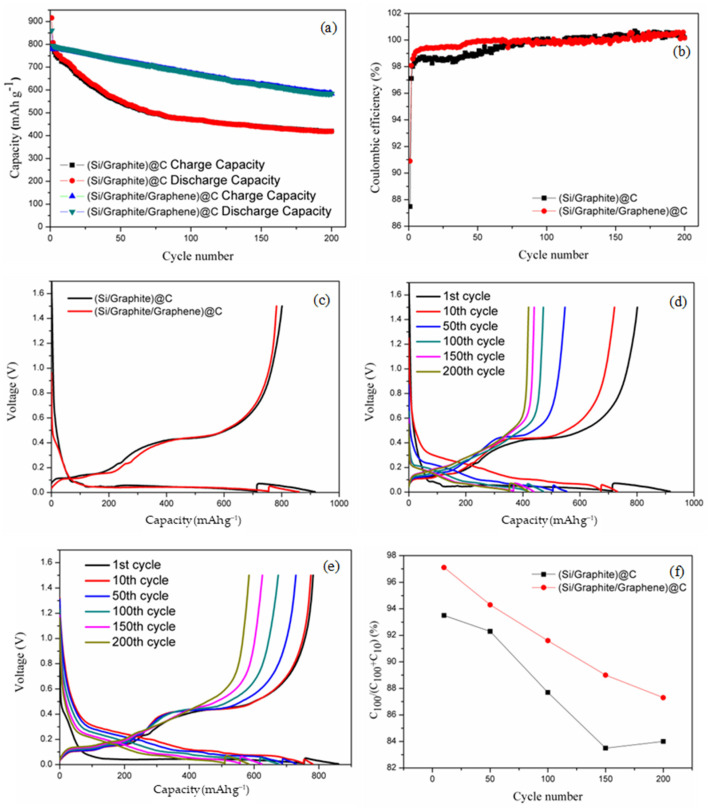
(**a**) Cycling performance, (**b**) first charge/discharge curves, and (**c**) first charge/discharge curves of (Si/graphite)@C and (Si/graphite/graphene)@C composite; discharge/charge curves of (**d**) (Si/graphite)@C composite and (**e**) (Si/graphite/graphene)@C composite; (**f**) capacity ratio variation curves of discharge current at 100 mA/g with different cycles.

The cycling performance and coulombic efficiencies of the obtained composites were evaluated, as shown in Figure 5a,b, respectively. The first discharge and charge capacities of (Si/graphite/graphene)@C are 860.4 and 782.1 mAh/g, respectively. The ICE is 90.9%, and reversible charge capacity is 584.2 mAh/g for 200 cycles. Subtracting the gram-specific capacity provided by silicon from the first charge-specific capacity of the negative electrode material, it is calculated that the gram-specific capacity provided by the carbon material is at least 105.31 mAh/g (782.1–676.79). Assuming that the capacity of the carbon material does not decay after 200 cycles, subtracting the gram-specific capacity provided by the carbon material from the reversible charge-specific capacity of the negative electrode material after 200 cycles yields that the gram-specific capacity provided by silicon after 200 cycles is 478.89 mAh/g. After 200 cycles, comparing the specific capacity of silicon before and after the cycle, the final capacity retention rate of silicon in the anode material is 70.76% (478.89/676.79). Silicon in the anode material has good capacity retention. This shows that by adding carbon material, silicon can maintain good cycling performance.

On the other hand, the first discharge and charge capacities of (Si/graphite)@C are 915.9 and 801.3 mAh/g, respectively, with an ICE of 87.5% and reversible charge capacity of 419.6 mAh/g, after 200 cycles. The ICE of (Si/graphite)@C was lower than that of (Si/graphite/graphene)@C. The nano-Si particles in (Si/graphite)@C undergo volume expansion during discharging and volume shrinkage during charging. This typically results in the loss of electrical contact of the embedded lithium ions and the repeated formation of SEI films, which leads to lithium depletion and irreversible capacity loss. Graphene can prevent the loss of electrical contact during the discharge/charge process, thus improving the ICE of (Si/graphite/graphene)@C. Furthermore, the stability of the anode material structure is improved due to the excellent mechanical properties of graphene, thus leading to superior cycle properties and coulombic efficiency of (Si/graphite/graphene)@C. Consequently, the capacity retention ratio of (Si/graphite)@C increases from 52.4% to 74.5% after 200 cycles with the addition of graphene. The first discharge/charge curve in Figure 5c shows the discharge/charge voltage plateau for (Si/graphite/graphene)@C due to lithiation/delithiation of the active material. In Figure 5d,e, a comparative analysis of the discharge/charge curves of (Si/graphite)@C and (Si/graphite/graphene)@C shows that the voltage at the discharge/charge plateau reduced/increased from the 10th cycle to the 200th cycle after the 1st cycle activation. This is mainly due to worsening electrical contact and increasing thickness of the SEI film. Moreover, from the 1st cycle to the 50th cycle, the charge voltage plateau at 0.4 V in (Si/graphite)@C shows a sharp decrease. This indicates that the capacity contribution of the nano-Si particles is decreased due to the loss of electrical contact of the nano-Si particles during the charge/discharge process, with an irreversible change in volume. The cycling performance of the samples was studied by first discharging to 0.01 V at 100 mA/g and then discharging to 0.005 V at 10 mA/g. The capacity ratio variation of discharge capacity at 100 mA/g and total discharge capacity can be used to evaluate discharge ability at high current. As shown in Figure 5f, the capacity ratios of (Si/graphite/graphene)@C and (Si/graphite)@C are 87.2% and 84.1%, respectively, after 200 cycles, and the capacity decrease rate of (Si/graphite/graphene)@C is slower than that of (Si/graphite)@C with an increase in the number of cycles, which suggests that the high current discharge ability and high rate-cycling stability of the former composite are higher than those of (Si/graphite)@C. The incorporation of graphene reduces electrochemical polarization, which improves high current discharge capability. Moreover, the sufficient mixing of graphene and silicon nanoparticles alleviates the uneven stress distribution caused by the volume expansion of silicon and improves the cycling performance of the anode material at high multiplicity.

Table 1 shows the electrochemical properties and material structures of several of our earlier-prepared silicon–carbon composite anode materials for lithium batteries. Compared to previous silicon–graphite anode materials, the addition of graphene resulted in a significant increase in the first coulomb efficiency of the materials. However, the excessive specific surface area of the carbon-based material is not favorable for the increase of the first coulomb efficiency [30]. This may be due to the fact that uniform mixing of graphene and nanosilicon affects the formation of the amorphous carbon layer, which makes the amorphous carbon layer smooth and leads to a decrease in the specific surface area of the composite material, which in turn reduces the formation of the SEI film and improves the first coulombic efficiency of the anode material.

### 3.3. Electrochemical Kinetic Properties

**Figure 6:**
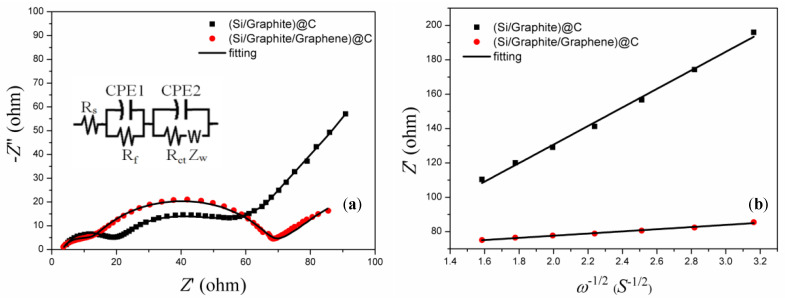
(**a**) Nyquist plots of graphite, Si/G composite, and (Si/G)@C composite after 10 cycles; (**b**) relationship between Z′ and ω^−1/2^ in low-frequency region derived from Figure 6a.

The electrochemical impedance curves of the (Si/graphite)@C and (Si/graphite/graphene)@C electrodes after 10 cycles, in a fully charged state, were fitted by an equivalent circuit shown in the right corner in Figure 6a to study the electrode structure change and electrochemical kinetics of the samples, and the results are listed in Table 2. Contact resistance R_s_ (including solution resistance between the working and reference electrodes)of the (Si/graphite/graphene)@C electrode is 3.39 Ω, which is lower than that of the (Si/graphite)@C electrode, indicating that the addition of graphene enhanced particle contact and improved electrical conductivity, leading to low contact resistance [33]. R_f_ reveals the forming condition of the SEI films. Since graphene has excellent mechanical properties, it promotes a stable SEI film [33,34]; thus, the R_f_ value of the (Si/graphite/graphene)@C electrode is 8.52 Ω, which is lower than that of the (Si/graphite)@C electrode. Furthermore, in (Si/graphite/graphene)@C, graphene can inhibit particle pulverization, preventing an increase in the charge transfer surface; thus, the charge transfer resistance, R_ct,_ of (Si/graphite/graphene)@C is higher than that of the (Si/graphite)@C [21,28].

The presence of a straight line in electrochemical impedance spectra typically reveals lithium-ion diffusion kinetics [35,36]. The effective lithium-ion diffusion coefficient can be evaluated by the following equation [37,38]:

(1) $$D=\frac{R^{2}T^{2}}{2A^{2}n^{4}F^{4}C^{2}\sigma^{2}}\;$$

(2) $$C=\frac{n}{V}=\frac{m/M}{V}=\frac{\rho V/M}{V}=\frac{\rho }{M}\;$$

where *R* is the gas constant; *F* is the Faraday constant; n is the number of electrons in the electron transfer reaction and has a value of 1; *T* is 298 K; C is the lithium-ion concentration, which can be calculated from the densities and molecular weights of materials synthesized by different methods, with a value of 3.19 × 10^4^ mol m^−3^, and the derivation process is shown in Equation (2); and σ is the slope of the straight line Z′~ω^−1/2^ as shown in Figure 6b. The lithium-ion diffusion coefficient of the (Si/graphite/graphene)@C electrode is 6.86 × 10^−13^ cm^2^ s^−1^, which is about 70 times that of the (Si/graphite)@C electrode, mainly because graphene improved lithium-ion transport properties and lowered the thickness of the SEI film, which is also beneficial to lithium-ion diffusion. The high lithium-ion diffusion coefficient is in good agreement with the good cyclability and reversible charge capacity of the (Si/graphite/graphene)@C composite.

## 4. Conclusions

The (Si/graphite/graphene)@C obtained by adding graphene to the Si/C composite anode material showed excellent electrochemical properties. First discharge/charge capacities of 860.4 and 782.1 mAh/g were obtained, with an ICE of 90.9% and a capacity retention of 74.5% after 200 cycles. The cycling and rate performance of the (Si/graphite/graphene)@C anode material improved effectively compared with (Si/graphite)@C. The incorporation of graphene into the Si/C composite reduced contact resistance, inhibited the fragmentation of the active material particles, and reduced the formation of SEI films. Graphene led to improved lithium-ion diffusion and excellent mechanical properties during charging. This study demonstrated that the proposed (Si/graphite/graphene)@C is a promising anode material for LIBs with high energy density requirements.

## Figures and Tables

**Table 1:** Electrochemical performance and material structure of our earlier-prepared silicon–carbon composite anode materials for lithium batteries.

Material Structure	First Discharge Capacity	Initial Columbic Efficiency	Reference
Graphite-loaded carbon-coated nanosilicon and carbon-coated graphite	460 mAh/g	89.8%	[31]
Si/G composites (graphite mixed with silicon nanoparticles)	703.8 mAh/g	84.95%	[32]
(Si/G)@C composites (graphite mixed with silicon nanoparticles and coated with sucrose-cracked carbon)	644.3 mAh/g	88.93%	[32]
(Si/graphite/graphene)@C	860.4 mAh/g	90.9%	This work
(Si/graphite)@C	915.9 mAh/g	87.5%	This work

**Table 2:** Kinetic parameters of electrodes after 10 cycling.

Samples	R_s_ (Ω)	R_f_ (Ω)	R_ct_ (Ω)	Slope	DLi+ (cm^2^ s^−1^)
(Si/graphite)@C	4.13	13.87	30.96	53.94	9.36 × 10^−15^
(Si/graphite/graphene)@C	3.39	8.52	55.74	6.30	6.86 × 10^−13^

## Data Availability

Data are contained within the article.
